# A clinically feasible multiplex proteomic immunoassay as a novel functional diagnostic for pancreatic ductal adenocarcinoma

**DOI:** 10.18632/oncotarget.15653

**Published:** 2017-02-23

**Authors:** Kian-Huat Lim, Emma Langley, Feng Gao, Jingqin Luo, Lin Li, Gary Meyer, Phillip Kim, Sharat Singh, Vladamir M. Kushnir, Dayna S. Early, Daniel K. Mullady, Steven A. Edmundowicz, Sachin Wani, Faris M. Murad, Dengfeng Cao, Riad R. Azar, Andrea Wang-Gillam

**Affiliations:** ^1^ Division of Oncology, Department of Internal Medicine, Washington University School of Medicine, St. Louis, MO, USA; ^2^ Division of Gastroenterology, Department of Internal Medicine, Washington University School of Medicine, St. Louis, MO, USA; ^3^ Department of Pathology and Immunology, Washington University School of Medicine, St. Louis, MO, USA; ^4^ Department of Surgery, Division of Public Health Sciences, Washington University School of Medicine, St. Louis, MO, USA; ^5^ Prometheus Laboratories Inc., San Diego, CA, USA; ^6^ Division of Gastroenterology and Hepatology, University of Colorado Anschutz Medical Campus, Aurora, CO, USA; ^7^ NorthShore University HealthSystem, Evanston, IL, USA

**Keywords:** pancreatic cancer, proteomics, fine-needle aspiration, signaling events, prognosis

## Abstract

To date, targeted therapy for pancreatic ductal adenocarcinoma (PDAC) remains largely unsuccessful in the clinic. Current genomics-based technologies are unable to reflect the quantitative, dynamic signaling changes in the tumor, and require larger tumor samples that are difficult to obtain in PDAC patients. Therefore, a highly sensitive functional tool that can reliably and comprehensively inform intra-tumoral signaling events is direly needed to guide treatment decision. We tested the utility of a highly sensitive proteomics-based functional diagnostic platform, Collaborative Enzyme Enhanced Reactive-immunoassay (CEER^TM^), on fine-needle aspiration (FNA) samples obtained from 102 patients with radiographically-evident pancreatic tumors. Two FNA passes were collected from each patient, hybridized to customized chips coated with an array of capture antibodies, and detected using two enzyme-conjugated antibodies which emit quantifiable signals. We demonstrate that this technique is highly sensitive in detecting total and phosphorylated forms of multiple signaling molecules in FNA specimens, with reasonable correlation of marker intensities between two different FNA passes. Notably, signals of several markers were significantly higher in PDAC compared to non-cancerous samples. In PDAC samples, we found high total c-Met signal to be associated with poor survival, and confirmed this finding using an independent PDAC tissue microarray.

## INTRODUCTION

To date, the prognosis for pancreatic ductal adenocarcinoma (PDAC) remains dismal. Complete surgical resection offers the only chance for cure, but is limited to a small fraction of patients who are diagnosed at early stage. Even then, most patients who undergo seemingly successful resection eventually succumb to disease relapse despite adjuvant treatment [1]. Underlying the aggressive nature of PDAC is a complex, deregulated signaling circuitry woven by several genetic alterations such as oncogenic mutations of *KRas*, overexpression of *EGFR/HER* family members, and loss of key tumor suppressors including *p53, CDKN2A and SMAD*4, which cooperatively enhance the survival of PDAC cells and resist the killing effect of therapies [2–5]. Although targeting these deregulated signaling events holds promise to improve the outcome of PDAC patients, clinical success remains limited [6]. The advent of next generation sequencing technologies has helped realize the goal of “personalized oncology” by allowing patients to be allocated to clinical trials tailored towards genomic alterations found in their tumors [7]. These techniques, despite being promising, have met with their own set of challenges, which include low tumor cellularity that is typical of PDAC, tumor heterogeneity, clonal evolution, and importantly, frequent discordance between genotype and cancer phenotype [8]. On the other hand, a “functional” diagnostic based on proteomics may serve as a useful complementary tool by providing the most direct link to the phenotype of cancer cells [9]. As most targeted agents are signaling modulators, a proteomics-based diagnostic that can inform the biological effect of targeted agents in real-time within the tumor will be extremely helpful in assessing treatment response, identifying potential resistance mechanisms and guiding further treatment decision, all of which are impossible using archived tumor samples.

In this study, we report the use of a multiplex proteomic-based assay, Collaborative Enzyme Enhanced Reactive-immunoassay (CEER^TM^), that is clinically feasible, highly sensitive and specific. This platform was previously shown to be successful in detecting rare “HER2-activated” circulating tumor cells in HER2-negative metastatic breast cancer patients [10], indicating its high sensitivity and ability to provide potentially actionable information beyond the genomic and transcriptomic levels. We show that the CEER™ platform allows simultaneous detection of the abundance and activation status of multiple key signaling molecules (or “markers”) that are uniquely deregulated in PDAC but not in normal tissues obtained from fine-needle aspirations (FNA), indicating high specificity and sensitivity. Encouragingly, while correlating the intensity of each marker to prognosis we found high c-MET signal in FNA specimens to be associated with poor prognosis, which is consistent with published literature based on immunohistochemistry of resected specimens, indicating great potential of this platform in studies of advanced, inoperable PDAC tumors in the future. Finally, we identified markers with previously unreported prognostic significance that may unveil novel understanding to PDAC biology.

## RESULTS

### Patient characteristics

**Figure 1:**
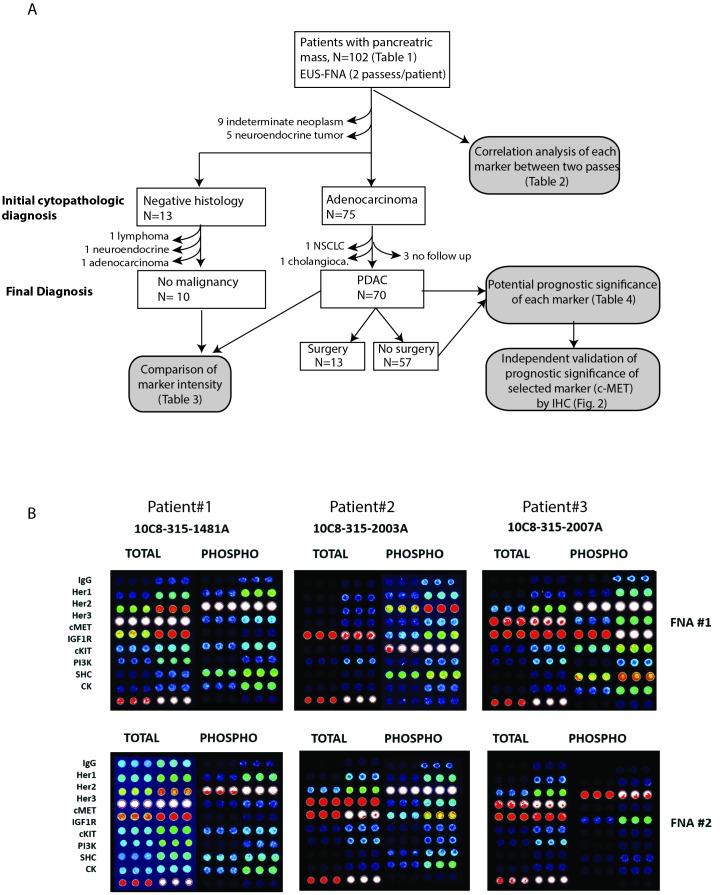
**A**. Flow diagram summarizing workflow of all analyses performed and reasons for inclusion and exclusion of subject for CEER studies. **B**. Representative images of CEER^TM^ arrays obtained from two FNA passes (#1 and #2) of three patients. Signal intensities ranges from dark (low) to bright white (high).

Between year 2010-2012, 102 patients with radiographic suspicion of pancreatic tumor underwent endoscopic ultrasonography (EUS) and fine-needle aspiration (FNA) of the primary pancreatic mass at Washington University School of Medicine and the affiliated Siteman Cancer Center (Figure 1A). Besides obtaining specimens needed to establish a histologic diagnosis, two additional FNA passes from the same tumor were collected for CEER^TM^ analysis. A cytological diagnosis of adenocarcinoma was initially made in 75 patients. However, one patient was later diagnosed with lung adenocarcinoma with pancreatic metastasis and another had extrahepatic cholangiocarcinoma with invasion to the pancreatic head. We excluded three other patients with PDAC who were lost to follow up from our institution. On the other hand, 13 patients had negative cytology, i.e. no evidence of malignant cells were detected from the initial cytological analysis. Three of these patients were later excluded after repeat biopsy showed malignancies including lymphoma, cholangiocarcinoma and pancreatic adenocarcinoma. Finally, five FNA samples showed neuroendocrine tumor and another nine were indeterminate due to insufficient tissue material. In summary, we focused our analyses on the 70 confirmed PDAC specimens with complete clinical follow up, and 10 specimens with negative (or non-cancerous) cytology (Figure 1A). These cytopathologic diagnoses of these ten negative samples were: 2 normal lymphoid content, 1 cystic content with bland epithelium, 5 benign pancreatic elements with reactive inflammatory elements, 2 non-diagnostic with scant cellularity.

Characteristics of all 102 patients and the 70 PDAC patients are provided in Table 1. For CEER^TM^ analysis, all FNA samples were collected and immediately processed to maximally preserve the phosphorylation status of each marker (Figure 1B). A total of 18 markers were assayed in the CEER^TM^ platform (see Material and Methods section and Table 2). All 70 patients were treated at Washington University School of Medicine and followed for up to five years or until death.

**Table 1:** Characteristics of patients enrolled into studies

Characteristics	All patients (N=102)	Confirmed PDAC, evaluable (N=70)
Median Age (range) ≤ 65 > 65	67.5 (38-89) 45 (44%) 57 (56%)	69.5 (38-89) 29 (41%) 41 (59%)
Gender (All) Male Female	53 (52%) 49 (48%)	34 (48.5%) 36 (51.5%)
Race Caucasian Non-Caucasian	89 (87.2%) 13 (12.8%)	60 (85.7%) 10 (14.3%)
Location of tumor Head Non-head	57 (55.9%) 45 (44.1%)	38 (54.3%) 32 (45.7%)
Tumor size (mm), median (range)	29 (17-70)	29 (13-70)
Final cytopathologic diagnosis PDAC Neuroendocrine Indeterminate Negative Other cancer	74 (72.5%) 6 (5.9%) 9 (8.9%) 10 (9.8%) 3 (2.9%)	
Initial Clinical Staging Resectable Borderline resectable/locally advanced Metastatic		12 (17.1%) 37 (52.9%) 21 (30%)
Treatment Surgery No surgery (chemo ± radiation)		13 (18.5%) 57 (71.5%)

**Table 2:** Spearman correlation coefficients of each marker between two passes

Variables	N	Spearman coefficient	P value
Total Cytokeratin	96	0.54005	<.0001
Phospho-HER1	99	0.59002	<.0001
Phospho-HER2	97	0.50394	<.0001
Phospho-HER3	91	0.53424	<.0001
Phospho-c-MET	96	0.48617	<.0001
Phospho-IGFR	56	0.49899	<.0001
Phospho-PI3K	85	0.33682	0.0016
Phospho-SHC	92	0.51318	<.0001
Total HER1	95	0.50101	<.0001
Total HER2	99	0.46615	<.0001
Total HER3	94	0.46471	<.0001
Total c-MET	96	0.56100	<.0001
Total IGFR	55	0.38793	0.0034
Phospho-AKT	84	0.61217	<.0001
Phospho-ERK1/2	76	0.45454	<.0001
Phospho-MEK	64	0.57802	<.0001
Phospho-RSK	64	0.47930	<.0001
Phospho-PRAS40	72	0.51943	<.0001
Phospho-RPS6	71	0.32136	0.0063

### Comparison of different FNA passes and between negative and positive PDAC samples

We first compared whether the signal intensities of each marker varied between the two FNA passes from each patient. As shown in Table 2, we observed modest to moderate degrees of consistency (Spearman's rho coefficients 0.32∼0.59) in the signal intensity between both passes across all markers. This result is highly reflective of the known low cellularity and potentially high intratumoral heterogeneity of PDAC tumors [3, 11], underscoring the need to obtain more than one pass for better representation of the whole tumor. To better represent tumor cell signaling, the FNA pass with higher level of cytokeratin (CK), a specific marker for epithelial cells [12], was used in subsequent analyses.

To determine specificity, we next compared the intensity of each marker between the 70 PDAC and 10 negative specimens (Table 3). First, we noted significantly higher CK signals in PDAC samples, compared to negative samples (*p* = 0.002), indicating presence of neoplastic epithelial content in PDAC samples. Remarkably, we also noted significantly higher signal intensities in several markers in PDAC samples. These include p-HER2 (*p* = 0.007), p-HER3 (*p* = 0.026), total HER2 (*p* = 0.006), total HER3 (*p* = 0.008), total c-MET (*p* < 0.001), total IGFR (*p* = 0.012), p-AKT (*p* = 0.046), p-ERK1/2 (*p* < 0.001) and p-PRAS40 (*p* = 0.043). In addition, a trend towards increased p-c-MET (*p* = 0.051) in PDAC was seen. Of these markers, total c-Met and p-ERK1/2 signals showed the strongest statistical difference between PDAC and negative samples (both *p* < 0.001), resonating previous reports that c-MET and p-ERK1/2 immunohistochemical staining to be markedly elevated in resected PDAC samples compared to normal pancreas [13–15]. In addition, the stronger p-ERK1/2 signal in PDAC samples is consistent with these kinases being key substrates that are activated downstream of mutant KRas protein, which is present in almost all PDAC [16, 17]. Overall, these results demonstrate that the CEER^TM^ technique is a robust, highly sensitive and specific tool that is capable of detecting salient signaling aberrations from the FNA specimens of PDAC patients.

**Table 3:** Comparison of overall marker intensities between negative vs PDAC specimens

Covariate	Signal Intensity, CU/ug (Mean ± SD, median)		P value
	Negative (N=10)	PDAC (N=70)	
Total Cytokeratin (CK)	116.76 ± 167.12, 47.15	536.93 ± 733.89, 354.25	**0.002**
Phospho-HER1	4.17 ± 5.76, 1.89	1.95 ± 2.65, 1.03	0.234
Phospho-HER2	0.33 ± 0.35, 0.14	2.78 ± 4.2, 1.15	**0.007**
Phospho-HER3	14.12 ± 14.84, 7.65	38.36 ± 45.71, 22.98	**0.026**
Phospho-c-MET	0.15 ± 0.16, 0.05	0.76 ± 1.46, 0.26	0.051
Phospho-IGFR	37.30 ± 47.26, 29.16	25.61 ± 45.06, 12.06	0.363
Phospho-PI3K	46.37 ± 44.40, 25.17	33.49 ± 32.5, 28.93	0.399
Phospho-SHC	3.23 ± 4.14, 1.84	5.56 ± 5.62, 4.21	0.200
Total HER1	54.38 ± 47.42, 42	61.44 ± 62.16, 40.25	0.765
Total HER2	1.22 ± 1.26, 0.55	10.18 ± 14.46, 3.9	**0.006**
Total HER3	54.72 ± 66.72, 25.0	387.96 ± 722.64, 120.2	**0.008**
Total c-MET	72.65 ± 66.73, 60.0	441.29 ± 649.53, 288.05	**<0.001**
Total IGFR	23.82 ± 15.67, 15.0	83.69 ± 108.82, 43.34	**0.012**
Phospho-AKT	0.80 ± 1.23, 0.26	3.25 ± 5.47, 1.36	**0.046**
Phospho-ERK1/2	0.92 ± 0.86, 0.88	7.8 ± 10.66, 4.78	**<0.001**
Phospho-MEK	0.36 ± 0.21, 0.43	1.77 ± 2.54, 0.74	0.120
Phospho-RSK	24.06 ± 17.89, 14.94	80.26 ± 80.7, 48.02	0.127
Phospho-PRAS40	12.61 ± 28.33, 1.0	41.6 ± 68.5, 9.24	**0.043**
Phospho-RPS6	50.13 ± 87.00, 4.84	18.74 ± 60.08, 1.58	0.377

### Prognostic significance of measured markers

Apart from having different intensities between PDAC and negative samples, we also noted wide patient-to-patient variation in intensities of all biomarkers, as discerned by the high standard deviations relative to the mean values of each marker (Table 3). Therefore, we hypothesize that the intensity of each marker, which represents the activity of various signaling pathways, may reflect the biology of the tumor and hence patient prognosis. Particularly, since the prognostic significance of some of these biomarkers, including c-Met, p-AKT and p-ERK1/2 have been published using immunohistochemical analyses of resected tumor samples, which arguably is the “gold standard” technique, we reasoned that being able to recapitulate similar findings using FNA specimens would, to some extent, validate the accuracy of this novel technique and substantiate its utility in future clinical application, particularly for most PDAC patients with inoperable disease where large amount of tumor specimen is not available. To this end, we followed all 70 PDAC patients for up to five years or until death. We divided all 70 PDAC patients into two survival groups (≤ or > median), using overall median survival (11.5 months, range 0.2 months ∼ 5 years) as a cut-off (Table 4). When markers between these two survival groups were compared, we found that tumors associated with poor survival had a strong trend towards having higher total c-MET level compared to those with better survival (*p* = 0.05), resonating previous report showing c-MET to be frequently overexpressed and represents an independent poor prognostic factor in resected PDAC patients [18, 19]. Interestingly, patients with poor survival had significantly lower levels of p-SHC (*p* = 0.002), p-PRAS40 (p = 0.002) and p-AKT (*p* = 0.025). Partly resonating this finding, another study also showed that high p-AKT IHC staining in resected PDAC samples was associated with better patient prognosis [20].

**Table 4:** Prognostic significance of age and tested markers in PDAC patients

Covariate	Statistics	All patients (N=70)			No surgery (N=57)		
		Survival ≤ median	Survival > median	P value	Survival ≤ median	Survival > median	P value
Age	Mean Median	70.9 71.6	65.1 64.9	0.055	72.4 71.8	64.5 64.8	**0.018**
Phospho-HER1	Mean Median	1.85 0.77	2.03 1.44	0.291	1.39 0.63	1.89 1.48	0.235
Phospho-HER2	Mean Median	3.07 0.81	2.49 1.81	0.379	2.66 0.74	2.37 1.68	0.442
Phospho-HER3	Mean Median	34.09 20.97	42.13 29.91	0.459	28.72 22.29	33.42 25.21	0.806
Phospho-c-MET	Mean Median	0.99 0.16	0.55 0.39	0.453	0.7 0.16	0.61 0.28	0.601
Phospho-IGFR	Mean Median	32.72 11.7	19.12 12.06	0.702	33.4 11.7	15.04 10.64	0.462
Phospho-PI3K	Mean Median	28.79 23.72	37.79 30.46	0.451	27.05 23.72	30.4 28.73	0.802
Phospho-SHC	Mean Median	3.83 2.03	7.15 6.42	**0.002**	3.03 1.83	6.66 6.17	**0.006**
Total HER1	Mean Median	64.88 47.41	58.19 35.37	0.839	61.05 47.41	46.29 33.51	0.390
Total HER2	Mean Median	9.78 2.93	10.55 5.8	0.309	8.72 2.55	8.88 4.42	0.396
Total HER3	Mean Median	455.48 106.7	326.22 129.2	0.779	433.26 113.05	240.94 102.35	0.728
Total c-MET	Mean Median	619.84 385.7	272.95 213.5	**0.050**	684.9 440.3	260.19 154.4	**0.027**
Total IGFR	Mean Median	92.94 41.5	74.97 45.17	0.807	86.39 41.5	57.72 39.53	0.396
Phospho-AKT	Mean Median	1.76 0.82	4.57 3.19	**0.025**	1.72 0.78	3.55 2.94	0.062
Phospho-ERK1/2	Mean Median	7.62 4.31	7.96 6.22	0.482	8.04 4.39	6.65 6.22	0.665
Phospho-MEK	Mean Median	1.36 0.53	2.12 1.1	0.318	1.24 0.54	1.68 1.01	0.701
Phospho-RSK	Mean Median	84.14 44.07	76.79 73.75	0.572	88.02 34.24	73.11 46.58	0.716
Phospho-PRAS40	Mean Median	22.03 2.94	59.5 31.26	**0.002**	21.12 3.16	42.87 13.73	**0.032**
Phospho-RPS6	Mean Median	20.31 1.26	17.35 1.58	0.475	24.28 1.26	16.64 1.52	0.911

Besides these biomarkers, clinical parameters such as clinical stage (including tumor size and lymph node status), tumor grade, age at diagnosis, medical comorbidities all have significant prognostic implication in PDAC patients. All these individual parameters culminate into a composite decision of surgical resection, which to date is the single most important prognostic factor in the outcome of PDAC patients [21]. Of all 70 PDAC patients we analyzed, patients who underwent curative resection (N = 13, or 18.6%) had a median survival of 20.8 months (range 5.1 months ∼ alive at 46 months) compared to 8.2 months (range 0.2∼33 months, all died) for those who could not be resected (N = 57, or 81.4%, Wilcoxon p = 0.003). Among these 70 patients, we did not detect statistically significant differences in survival based on age, tumor size, nodal status, stage, or tumor grade, probably due to small sample size and the fact that most patients were diagnosed at inoperable stages. Of the 57 non-operable patients, patients with locally-advanced (N = 37) or metastatic disease (N = 20) had a median OS of 9.7 and 6.7 months respectively, which did not reach statistical significance (Wilcoxon P = 0.36) probably due to small sample size and the inherent aggressive nature of this disease.

We next explored the prognostic impact of each marker among the inoperable patients (N = 57), which is rarely reported in the literature due to the limited availability of PDAC tissues from these patients. By focusing on these patients, we believe we could more accurately capture the biological role of each marker in the natural progression of PDAC without the dramatic interference of surgical removal. Therefore, any markers later found to have prognostic impact could have a larger therapeutic implication in the future as most PDAC patients are either diagnosed at, or eventually will progress to, advanced stage. To this end, we divided all 57 patients into two groups using median OS (8.2 months) as a cut-off, and compared the intensity of all markers between these two groups (Table 4). Again, we found that patients will poorer prognosis had significantly higher expression levels of total c-MET (p = 0.027). Interestingly, these patients also had significantly higher levels of p-SHC and p-PRAS40 (p = 0.006 and p = 0.032, respectively). Importantly, patients with poorer prognosis were significantly older (mean 72.4 years old) compared to those with better prognosis (mean 64.5 years old, p = 0.018), consistent with published literature showing older age as a poor prognostic marker [22].

### Independent validation of prognostic significance of c-MET using PDAC tissue microarray

**Figure 2:**
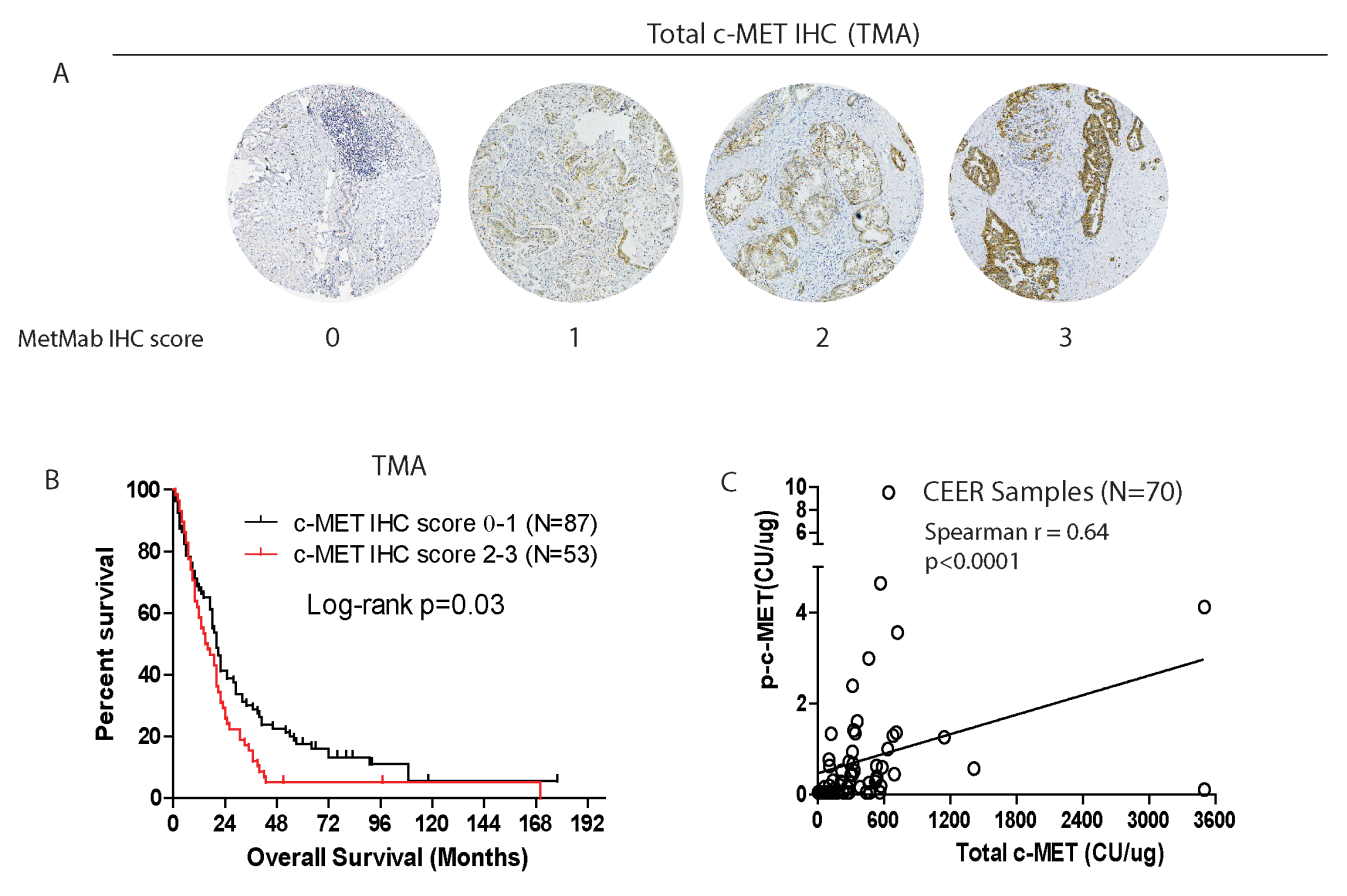
Independent validation of prognostic significance of total c-MET IHC staining in PDAC tissue microarray from another cohort of 140 patients **A**. Representative IHC images showing various assigned intensity of total c-MET staining in PDAC tissue microarray. **B**. Kaplan-Meier survival analysis of patients based on c-MET IHC score. **C**. Spearman correlation between total and phospho-c-MET signals of FNA samples subject to CEERTM.

While the robust quantitative capability of the CEER^TM^ platform has enabled prognostication to be ascribed to certain markers such as total c-Met, p-SHC and p-PRAS40, further validation is needed. First, high level of c-MET immunohistochemical staining in resected PDAC tumors has already been reported by different groups to be a poor prognostic marker [18, 19]. Second, to further confirm this finding, we performed c-MET IHC staining on an independent PDAC tissue microarray built at our institution. Using the MetMab visual IHC scoring criteria (0 to 3+) that is widely adopted [23, 24] (Figure 2A), we found that the overall expression of c-MET is high (MetMab IHC score 2+ or 3+) in 53 out of 140 samples (or 37.9%) of all PDAC samples. Consistent with our findings from the CEER^TM^ platform, high c-MET IHC score is associated with poor prognosis in this independent cohort of patients (p = 0.03) (Figure 2B). On further analysis of PDAC samples, we indeed found significant correlation between the intensities of total and phospho-c-MET signals (Spearman r 0.64, p < 0.0001; Figure 2C), further supporting targeting c-MET to improve the outcome for PDAC patients. Overall, our results further substantiate the quantifying power of the CEER^TM^ platform, rendering it extremely useful in future clinical trial design.

## DISCUSSION

Development of a powerful “functional” diagnostic is direly needed to monitor treatment response in the era of targeted therapy. The described CEER^TM^ platform is particularly powerful in many aspects. First, this platform provides, in addition to expression levels, activation status of multiple key signaling molecules, which is impossible with current transcriptomic or genomic techniques. In PDAC, inhibitors that target the key KRas effectors such as the Raf-MEK-ERK and PI3K-AKT-mTOR pathways are being actively pursued in clinical trials, albeit with little success due to rapid emergence of various different resistance mechanisms including kinome reprogramming [26]. While these mechanisms can be readily identified, and overcome using cultured cells in the lab, being able to do so in the clinic in real time is essential in initial clinical trial allocation, and informing combinatorial strategies if no response is seen. To this end, the CEER^TM^ technique could potentially provide a comprehensive, quantifiable signaling changes across multiple signaling pathways within the tumor tissues before and after therapeutic intervention. Second, the ultra-sensitivity of this platform allows analysis to be performed using scant amount of tissues such as FNA samples, which is especially attractive for clinical trials on pancreatic cancer, where tissues are limited and prone to degradation by catalytic enzymes in the pancreas. Third, the CEER^TM^ platform provides clear proteomic distinction between normal pancreatic and PDAC tissues, indicating robust specificity. For instance, significantly higher signal intensity of EGFR/HER signaling, total c-MET, total IGFR, p-AKT, p-ERK1/2 and p-PRAS40 were seen in PDAC compared to non-cancerous samples. Of these markers, enhanced phosphorylation of ERK1/2, AKT and PRAS40 are all known events driven by the KRas oncoprotein. Therefore, these markers could serve as readouts in clinical trials for various inhibitors against the KRas effectors. Forth, processing of tumor specimens and data analysis are relatively less sophisticated than genomic or transcriptomic tools, which will diminish both cost and turnaround time. In summary, all these advantageous features render the CEER^TM^ technique an extremely attractive companion diagnostic in future clinical trial design.

An important finding from analyzing the result from CEER^TM^ is the wide patient-to-patient variability in the signal intensity of each marker. This finding is reflective of the highly heterogeneous biology of PDAC, as supported by discovery of various molecular subtypes of PDAC based on genomic and transcriptomic analyses [2–4]. On this basis, the CEER^TM^ platform may be able to provide essential complementary proteomic data that could aid treatment selection and even prognostication. Supporting this notion, we showed that high level of c-MET expression by CEER^TM^ is associated with inferior survival for patients with advanced inoperable PDAC. We validated this data by immunohistochemistry, which is the most widely accepted technique. In addition, the poor prognostication of high c-MET level in PDAC has also been published in retrospective analysis [19], and preclinical studies also show that activation of c-MET in PDAC can enhance proliferation, survival, invasiveness, and treatment resistance [14, 25]. Several c-MET inhibitors are being evaluated in clinical trials for various cancer types including PDAC, with promising results still lacking, presumably due to poor patient selection and de novo resistance [27]. Biomarkers to predict response such as circulating HGF or c-MET levels, c-MET genomic amplification or protein overexpression are being assessed. To this end, the CEER^TM^ technique could certainly provide pre-treatment levels of total and activated c-MET to aid patient selection, and importantly, assessment of on-target effect and potential escape mechanisms at treatment failure.

Intriguingly, our data showed that high phosphorylation of SHC and PRAS40 proteins to be associated with better prognosis in PDAC patients, which have not been reported. In contrast, high p-SHC correlates with aggressive features and poor prognosis in gastric and breast cancer [28, 29], which underscores caution when extrapolating research data from one cancer type to another. The SHC proteins consists of three different splicing isoforms, p46shc, p52shc and p66shc, and are all members of the Src homologous- collagen homologue adaptor protein family that have very divergent roles in regulating signal downstream of growth factor receptors [30]. Since the antibody used in our CEER^TM^ platform recognizes all three SHC isoforms, development of isoform-specific phospho-SHC antibodies will be invaluable in informing the prognostic implication of each isoform and provide novel therapeutic opportunities. Similarly, the association of higher p-PRAS40 with better survival in PDAC is also different from a study in gastric cancer, where presence of p-PRAS40 was associated with aggressive histologic features and poor prognosis [28]. The proline-rich AKT substrate of 40-kDA (PRAS40) is a substrate of AKT which functions as an inhibitor to mTORC1 complex in regulating glucose metabolism [31, 32] . To date, the role of PRAS40 in PDAC is largely unclear and should be investigated. Overall, these interesting observations underscore the ability of the CEER^TM^ in revealing, in an unbiased manner, novel and potentially important clues that can be pursued.

There are a few limitations in our study. First, low tumor cellularity and tumor heterogeneity remain concerns especially in PDAC. Although we focused our analyses on the FNA sample that has the higher CK value, signals from stromal and immune cells may interfere with the readouts we obtained. In future studies, more FNA passes should be obtained for analysis of the sample with the highest CK value. Second, we are unable to determine the prognostic impact of different chemotherapeutic regimens on patient survival due to small patient number (N = 57). However, all patients received systemic treatment per the NCCN guidelines, which include 5-FU or gemcitabine-based chemotherapy regimens as standard-of-care or part of clinical trials. Larger studies in the future are needed to confirmed the prognostic impact of individual markers examined in our present study.

In conclusion, we demonstrate, for the first time in literature successful utilization of an unbiased, proteomic-based functional diagnostic that allows comprehensive elucidation and quantification of signaling aberrations in primary PDAC tumors. We demonstrate that this technique is clinically feasible, highly sensitive, specific and reliable. In the current era of precision oncology when the use of kinase inhibitor is increasingly frequent, this technique could provide irreplaceable proteomic information within the tumor, which could be critical in assessing the treatment effect and providing secondary signaling changes that could inform resistance mechanisms. As such, we believe this technique has irreplaceable value, in complement to genomic and transcriptomic tools, in realizing the goals of personalized oncology.

## MATERIALS AND METHODS

### Tumor specimen procurement

Tumor specimens were prospectively collected *via* endoscopic ultrasound-guided fine needle aspiration (EUS-FNA) from 102 patients presenting with a suspicious pancreatic mass as summarized in Figure 1A. Informed consent, specimen collection and future data collection were all conducted under IRB approval (Washington University in St. Louis IRB protocol #201106347). In addition to the passes obtained for routine clinical care, two EUS-FNA passes were performed and immediately collected in separate vials containing 100 μl of preservation and lysis solution (proprietary information, Prometheus Laboratories Inc.). The resulting lysates were shipped to Prometheus Laboratories (San Diego, CA) at ambient temperature within 48 hours of sample collection, and stored at -70°C upon receipt until subsequent CEER^TM^ analysis.

### Collaborative enzyme enhanced reactive immunoassay (CEER^TM^)

To measure the expression and activation levels of receptor tyrosine kinases and signal transduction proteins in clinical specimens, we employed CEER^TM^, a highly sensitive multiplexed immunoarray platform. Detailed methods for this technology have been described previously [1–5]. Briefly, capture antibodies (Abs) were printed on nitrocellulose-coated glass slides (ONCYTE^R^ Grace Biolabs) using a non-contact printer (Nano-Plotter, GeSiM). The spot diameter was approximately 175 μm and printed slides were kept in a desiccated chamber at room temperature. Capture Abs were printed in triplicate and at serial dilution concentrations of 1 mg/mL and 0.5 mg/mL. Purified mouse-IgGs served as negative controls. Immunoarray slide configurations and assay format were performed as previously described [1–5]. Briefly, immunoarray slides were rinsed with TBST (50 mM Tris/ 150 mM NaCl/ 0.1% Tween-20, pH 7.2-7.4) and blocked for 1 hour at room temperature (RT). Serially diluted lysate controls in 80 μL dilution buffer (2% BSA/ 0.1% TritonX-100/ TBS, pH 7.2-7.4) and samples were added to designated sub-arrays on slides, then incubated overnight at RT. After several washes, slides were incubated with two detector Abs (for different epitopes) conjugated with glucose oxidase (GO) and horseradish peroxidase (HRP) respectively for 2 hours at RT. After washing slides with TBST to remove unbound detector Abs, GO/HRP-mediated tyramide signal amplification process was triggered by adding biotin-tyramide solution and incubating for 30 mins. Local deposition of biotin-tyramide was detected by incubation with streptavidin-Alexa Fluor647 (Life Technologies, Carlsbad, CA) for 40 min. Slides were washed with TBST, dried and immediately processed on a high-resolution fluorescence microarray scanner (PowerScanner, Tecan). Background-corrected signal intensities were averaged for capture antibodies printed in triplicate. For each marker, a standard curve was generated from serially diluted control lysates prepared from specific cell lines. HCC827, a NSCLC adenocarcinoma cell line carrying *EGFR* gene amplification and exon 19 deletion, was used for EGFR and MET quantifications, while breast cancer cell lines BT474 and T47D, served for HER2/PRAS40/RPS6 and HER3/IGF1R/PI3K/CK quantifications respectively. Alternatively, standard curves were generated from serially diluted recombinant proteins for AKT, ERK, MEK and RSK assays. Each curve was plotted as a function of signal intensity measured as relative fluorescence unit (RFU) vs. log concentration of cell lysates/ recombinant proteins or Computed Unit (CU). The data were fit to a five-parameter equation by nonlinear regression, simultaneously fitting both dilutions of the capture Ab as described previously [10, 33]. CEER measurements are determined in Computed Unit (CU). CU is a representation of marker expression/ activation in unknown samples relative to that of control cell lines with known expression /activation levels. Hence, a sample with 1 CU of HER1 expression has an RFU value equivalent to 1 standard reference HCC827 cell. Because expression and activation of each marker is determined in unique CEER™ assays with different cell line standards, only CU values of the same marker across various samples can be compared.

### Pancreatic cancer TMA, c-Met immunohistochemistry (IHC) and scoring

The PDAC TMA was constructed from FFPE surgical specimens archived at the Department of Pathology and Immunology at Washington University under IRB approval and was previously published [34, 35]. Complete treatment history, clinical follow up and outcomes were available for all patients. For total c-MET staining, antigen-retrieval was performed by incubation in 0.01 mol/L citrate buffer (pH 6.0) and heating in a pressure cooker. Sections were incubated at 4°C overnight with total c-MET antibody (D1C2, Cell Signaling Technology, 1:200), followed by staining using the DAB Peroxidase (HRP) Substrate Kit with Nickel (Vector Laboratories cat#SK-4100). Stained slides were digitalized and scored independently by a GI pathologist (D.C) in blinded fashion using the MetMab IHC scoring method as published [23, 24]. Briefly, stained tumors were scored between 0-3+ based on : 0: no staining or < 50% of tumor cells with any intensity; 1+: ≥ 50% of tumor cells with weak or higher intensity but < 50% with moderate or higher intensity; 2+: ≥ 50% of tumor cells with moderate or higher intensity but < 50% with strong intensity; 3+: ≥ 50% of tumor cells staining with strong intensity).

### Statistical analysis

Due to the presence of detecting limits, all the measured markers were analyzed using non-parametric tests which were based on relative ranks rather than absolute values. The correlation between two FNA passes from the same patients were assessed using Spearman correlation coefficient. In subsequent analysis, the FNA pass with higher cytokeratin (CK) level was retained for data analysis. The difference in each marker between positive and negative PDAC samples was compared using Mann-Whitney rank-sum test. Among these 70 patients with positive PDAC, only 2 patients (who happened to have the longest follow-up times) were censored and the survival times were observed in all other patients. To assess the prognostic value of each marker on survival, the patients were categorized into 2 groups by median survival time, and the between-group difference in each marker was then compared using Mann-Whitney rank-sum test. The survival curves by other clinical characteristics such as stage and grade were also estimated using Kaplan-Meier product limit method. Since almost all the patients died during follow-up and the estimated survival curves were more likely to converge eventually, the difference between survival curves were compared by Gehan-Breslow-Wilcoxon (Wilcoxon) method (which is less sensitive to the proportional hazard assumption). All the tests were two-sided with p-value of 0.05 indicating significance. The data was analyzed using the standard package of SAS (Version 9.3, SAS Institute, Cary, NC).
